# Electron beam-based direct writing of nanostructures using a palladium β-ketoesterate complex

**DOI:** 10.3762/bjnano.16.41

**Published:** 2025-04-15

**Authors:** Chinmai Sai Jureddy, Krzysztof Maćkosz, Aleksandra Butrymowicz-Kubiak, Iwona B Szymańska, Patrik Hoffmann, Ivo Utke

**Affiliations:** 1 Empa - Swiss Federal Laboratories for Materials Science and Technology, Thun, Switzerland; 2 École Polytechnique Fédérale de Lausanne (EPFL), Lausanne, Switzerland; 3 Present address: Univ Rennes, CNRS, IPR (Institut de Physique de Rennes) - UMR 6251, Rennes, France; 4 Faculty of Chemistry, Nicolaus Copernicus University in Toruń (NCU), Toruń, Poland

**Keywords:** 3D nanoprinting, electron-induced molecule dissociation, focused electron beam-induced deposition, metal nanostructures, metalorganic complexes

## Abstract

Gas-assisted focused electron beam-induced deposition (FEBID) as a direct, minimally invasive 3D nanopatterning tool offers many advantages in making nanostructures with complex shapes and novel compositions for evolving nanotechnological applications. In this work, structures were nanoprinted using a fluorine-free β-ketoesterate complex, bis(*tert*-butylacetoacetate)palladium(II), [Pd(tbaoac)_2_]. The internal nanostructure and composition of the deposits were determined, and possible volatile products produced under electron-induced dissociation, explaining the composition, are investigated. A method to eliminate the residual gas contamination during FEBID was implemented. [Pd(tbaoac)_2_] contains large organic ligands and only about 5 atom % palladium in the pristine molecule, yet the obtained palladium content in the deposits amounts to around 30 atom %. This translates to an exceptional removal efficiency of about 90% for the ligand-constituting elements carbon and oxygen through electron-induced dissociation and desorption mechanisms. Comparison with other precursors confirms that the β-ketoesterate family has the highest ligand removal percentage and constitutes thus an interesting model chemistry for further high-metal-content FEBID studies. The possibility of growing nanopillars makes this complex a promising precursor for nanoprinting 3D structures with finely focused electron beams.

## Introduction

Direct fabrication of nanostructures without the use of masks, achieving scales down to a few nanometers with various patterns and shapes, offers significant advantages for a wide range of technological applications. These include areas that require plasmonic [1–3], phononic [3–4], magnetic [5–6], optoelectronic [7–9], and mechanical [10–12] properties at the nanometer scale. One method that is capable of creating such nanostructures is focused electron beam-induced deposition (FEBID) [13–23]. In this technique, a focused electron beam decomposes adsorbed molecules on a substrate in vacuum, resulting in a localized deposit at the irradiated area. When the precursor is delivered to the substrate in its gaseous form through a gas injection system (GIS), the process is termed as gas-assisted FEBID [23], commonly called FEBID. Variants such as liquid FEBID [24] and cryo-FEBID [25] also exist, though they follow a two-step process similar to lithography techniques [26]. Since the dissociation of molecules under an electron beam on the substrate can follow multiple pathways, the deposition parameters can greatly influence the composition, leading to the creation of novel materials [27]. Nanostructures produced by these methods also serve as prototypes for fundamental experiments on how material properties vary with dimension [28–29].

A key challenge with the FEBID technique is achieving pure metallic nanostructures [30]. To obtain metal deposits, metal complexes with organic and inorganic ligands are typically used as a metal source. Metalorganic precursors are preferred because of their vacuum stability and volatility. Achieving pure metal deposits requires the complete dissociation of all ligands from the metal atom and their desorption without producing further nonvolatile fragments under electron impact or getting trapped by newly adsorbing precursor molecules or diffusing molecules. The deposition process is governed by dissociation, desorption, adsorption, and diffusion mechanisms, all of which are influenced by experimental parameters [31] such as substrate temperature, molecular and electron flux, beam scanning strategies, and the choice of substrate and precursor. Altering any of these parameters can affect multiple deposition mechanisms simultaneously. The dissociation of precursors under electron impact is particularly complex because of the involvement of a broad spectrum of electron energies, including secondary electrons (SEs) and backscattered electrons (BSEs). This has a significant impact on the resulting composition, which depends on the specific precursor used. To produce pure metal structures, new precursors are being explored for FEBID applications.

In this study, we present a detailed analysis of the characteristics of deposits obtained using the new precursor bis(*tert*-butylacetoacetate)palladium(II), [Pd(tbaoac)_2_], a member of the β-ketoesterate complex group. Given the growing interest in carbon nanotubes (CNTs) and graphene for semiconductor applications [32–34], palladium is an important metal as it is the optimal material to make metallic contacts with CNTs [35]. Palladium nanoparticles are also being explored for biomedical applications and sensors [36]. Therefore, Pd nanoprinting via FEBID could emerge as a key technique for creating nanostructures for advanced technologies. So far, a few Pd-containing precursors including allyls [37] and β-diketonates [37–39] as ligands have been used in FEBID, yielding deposits with less than 30 atom % Pd. In this work, we report the structure and composition of deposits from [Pd(tbaoac)_2_] and discuss the deposition regimes involved. We also investigate the potential volatile products produced under electron impact on adsorbed [Pd(tbaoac)_2_] that could be responsible for the composition of the FEBID deposit and provide a comparison with previous results using other Pd precursors [37] and [Cu(tbaoac)_2_] [40–41]. The potential of growing 3D nanostructures with this precursor was also explored. The insights gained from this research could be valuable in the development of precursors tailored for FEBID.

## Experimental

**Figure 1:**
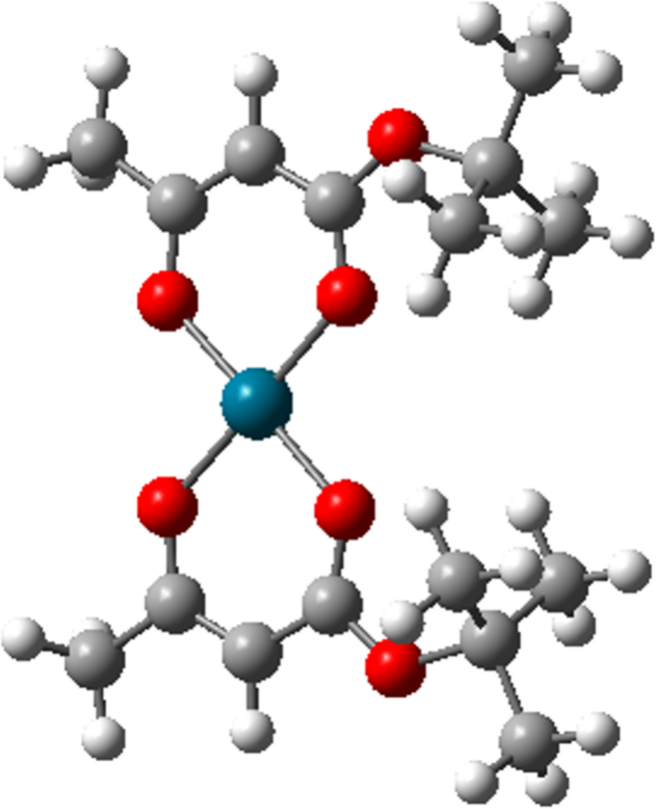
The structure of bis(*tert*-butylacetoacetate)palladium(II) precursor. The atom color labeling is white: H, grey: C, red: O, and bluish green: Pd [42].

The structure of [Pd(tbaoac)_2_] is shown in Figure 1. The precursor was synthesized following the method outlined by Butrymowicz and colleagues [42]. FEBID was performed using a Hitachi S3600 scanning electron microscope (SEM), which features a high-vacuum chamber and a tungsten filament electron source. Native-oxide Si(100) was employed as a substrate, which was thoroughly cleaned with multiple cycles of water–acetone–isopropanol solvents in an ultrasonification bath and then dried using pressurized N_2_ gas. For the precursor delivery, we used a heatable, custom-built GIS. Before precursor filling, the GIS underwent the same cleaning procedure as the substrate. The solid precursor was introduced into the GIS under ambient conditions as it was tested stable. The GIS nozzle was placed 200 µm above the substrate at an angle of 30° to the substrate plane.

Both GIS and substrate were heated using resistive heating wires. FEBID was carried out at an operating pressure of 2.0 × 10^−6^ mbar. For all deposits, the GIS and substrate temperatures were set to 85 and 70 °C, respectively. At these temperatures, no thermal decomposition occurred inside the GIS, nor did condensation or CVD processes take place on the substrate, while practical growth rates of deposits were observed. The precursor flow rate in the deposited area was approximately about 2 × 10^16^ molecules·s^−1^ or 3.7 × 10^3^ molecules·nm^−2^·s^−1^. This was determined through measuring the difference in weight of the precursor before and after experiments inside the GIS. This is the amount of precursor consumed for the experimental duration. A 20 keV electron beam was used with a probe current of 0.7 nA, as measured on a Faraday cup using a Keithley picoamperemeter. This corresponds to a current of 0.5 nA on the native-oxide Si(100) substrate, with the remaining 0.2 nA attributed to BSEs and SEs emitted from the substrate. The electron arrival rate was 3 × 10^10^ electrons·s^−1^ or 4 × 10^3^ electrons·nm^−2^·s^−1^. A XENOS pattern generator and ECP software were used to perform the square depositions with spiral inward scanning strategy. In particular, a 5 × 5 µm^2^ square deposit was created with a 3 nm pitch, 500 ns dwell time (100 µs effective dwell time with a 600 nm FWHM of the electron beam), and 2000 cycles.

For deposit morphology observation, a high-resolution Hitachi S4800 FESEM was used. The chemical composition of the deposits was confirmed through energy-dispersive X-ray spectroscopy (EDX) using a silicon drift detector from Oxford Instruments. EDX was performed with 6 keV electron beam at 500 pA, and the signals were collected for 60 s. Atomic force microscopy (AFM) measurements were conducted on an NT-MDT NTEGRA Spectra system, and data was analyzed using Gwyddion and Origin software. To accurately obtain the composition of the thin focused electron beam (FEB) deposit containing Pd, C, and O without substrate interference (native silicon oxide and silicon), the SAMx Stratagem thin film correction software, based on the work by Pouchou and Pichoir [43] was employed. Stratagem needs *k*-ratios (i.e., the ratio of X-ray intensity of a particular element of the sample under study to a tabulated standard for the same element) from EDX quantification software as input (in our case, Oxford Aztec), which were generated assuming isotropy (i.e., even distribution of all measured elements in the electron excitation volume). Using these *k*-ratios, Stratagem then recalculates the composition for a thin film (multi)layered structure. The native-oxide Si(100) substrate was accounted for by including a 1 nm thick SiO_2_ layer of density 2.65 g·cm^−3^ between the Si substrate and the deposit for the thin film correction. This way, the oxygen signal of the native oxide layer is separated from the signal of oxygen from ligand residues of [Pd(tbaoac)_2_] in the deposit.

Nanostructural observations were performed with transmission electron microscopy (TEM) using a probe-corrected ThermoFisher Scientific Titan Themis 200 G3 operating at an accelerating voltage of 200 kV. For this purpose, the FEB deposits were prepared on an ultrathin carbon support layer of less than 3 nm thickness supported by a lacey carbon membrane (PELCO) on a TEM grid. The TEM grid was fixed to the heatable stage. The deposition process was carried out in a Philips XL30 SEM microscope with the same temperatures and GIS positions as those employed for FEB depositions on native-oxide silicon substrates. A square area of 0.97 × 0.97 µm^2^ was deposited at 20 keV electron energy, with a dwell time of 500 ns and a point-to-pitch of 9.5 nm. The stage current was measured to 0.35 nA. The operating pressure was 1.5 × 10^−5^ mbar.

## Results and Discussion

**Figure 2:**
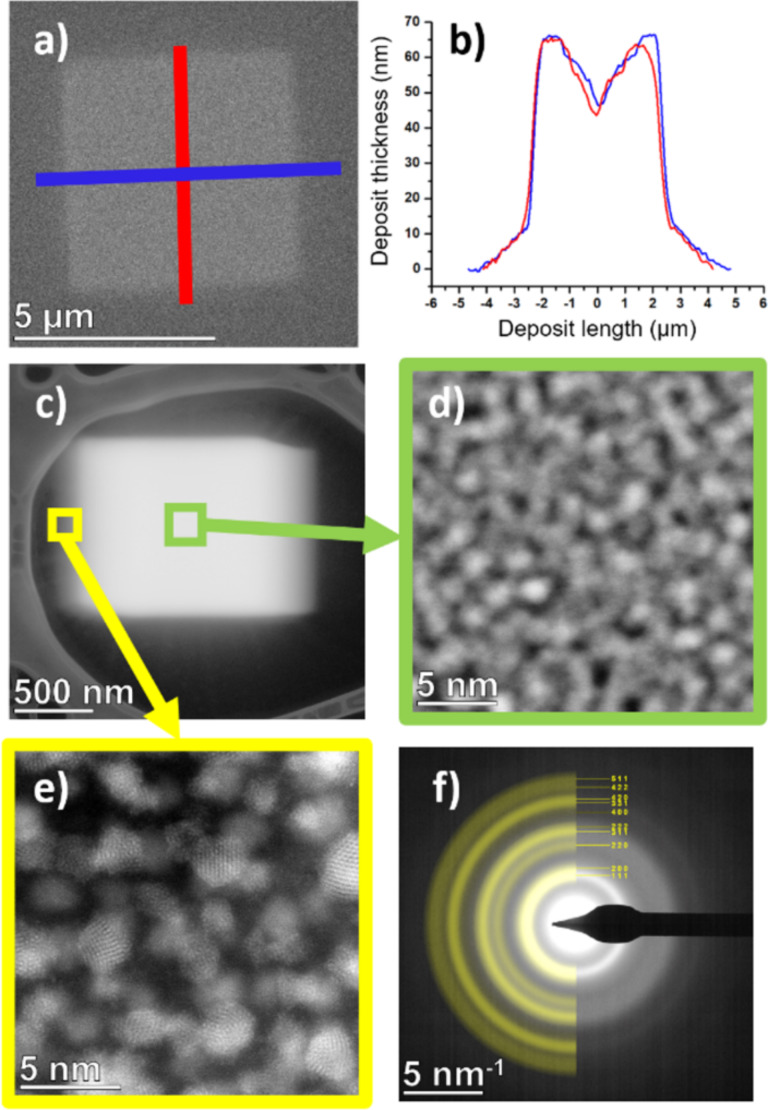
Characteristics of FEB nanoprinted square deposits with [Pd(tbaoac)_2_]. (a) Secondary electron SEM image of the FEB deposit on a native-oxide Si substrate with indicated AFM scan lines. (b) AFM thickness profiles along vertical and horizontal directions. (c) STEM image of the FEB deposit on a carbon membrane. (d) High-resolution STEM image from the center of the deposit. (e) High-resolution STEM image from the edge of the deposit. (f) SAED pattern from the edge of the deposit. Enlarged version of the images in panels (e) and (f) can be found in Supporting Information File 1, section S1 along with an additional STEM image.

The square deposit, fabricated on the native-oxide Si(100) substrate using a spiral inward scanning strategy, was analyzed to assess its morphology and structure. Secondary electron detection in SEM imaging reveals the topography and overall uniformity of the deposit (Figure 2a). The thickness profile extracted from the AFM data (Figure 2b) shows a dip at the center of the deposit. This shape is typical of the spiral scanning method when the rate of adsorbate dissociation exceeds the rate of adsorbate supply. The resulting geometry is primarily due to surface diffusion processes and directional gas flux adsorption [44–45]. No halo region was observed. The volume growth rate was approximately 0.032 µm^3^·min^−1^ or 7.62 × 10^−4^ µm^3^·nC^−1^, with a vertical growth rate of 0.02 nm·s^−1^.

For the STEM analysis, a square deposit was prepared on an ultrathin carbon support film spanning a lacey carbon membrane (Figure 2c). The deposit appears smeared because of drift caused by charging effects during the deposition process. High-resolution STEM imaging (Figure 2d,e) revealed a granular nanostructure with nanograins of around 2 nm in size. The thinner region of the FEB deposit, located at the edge of the deposit, reveals the crystalline structure of the grains (Figure 2e), where atomic columns can be observed for grains that were oriented along crystallographic directions. The crystalline structure was further examined using selected area electron diffraction (SAED). The resulting diffraction pattern (Figure 2f) shows diffuse diffraction rings characteristic of nanocrystalline materials. Indexing of the diffraction pattern confirmed the structure as metallic palladium (ICSD code 64918), matching the simulated pattern for a nanocrystalline material with a grain size of 2 nm. These results confirm that the FEBID material derived from [Pd(tbaoac)_2_] consists of metallic palladium nanograins embedded in a carbonaceous matrix.

**Figure 3:**
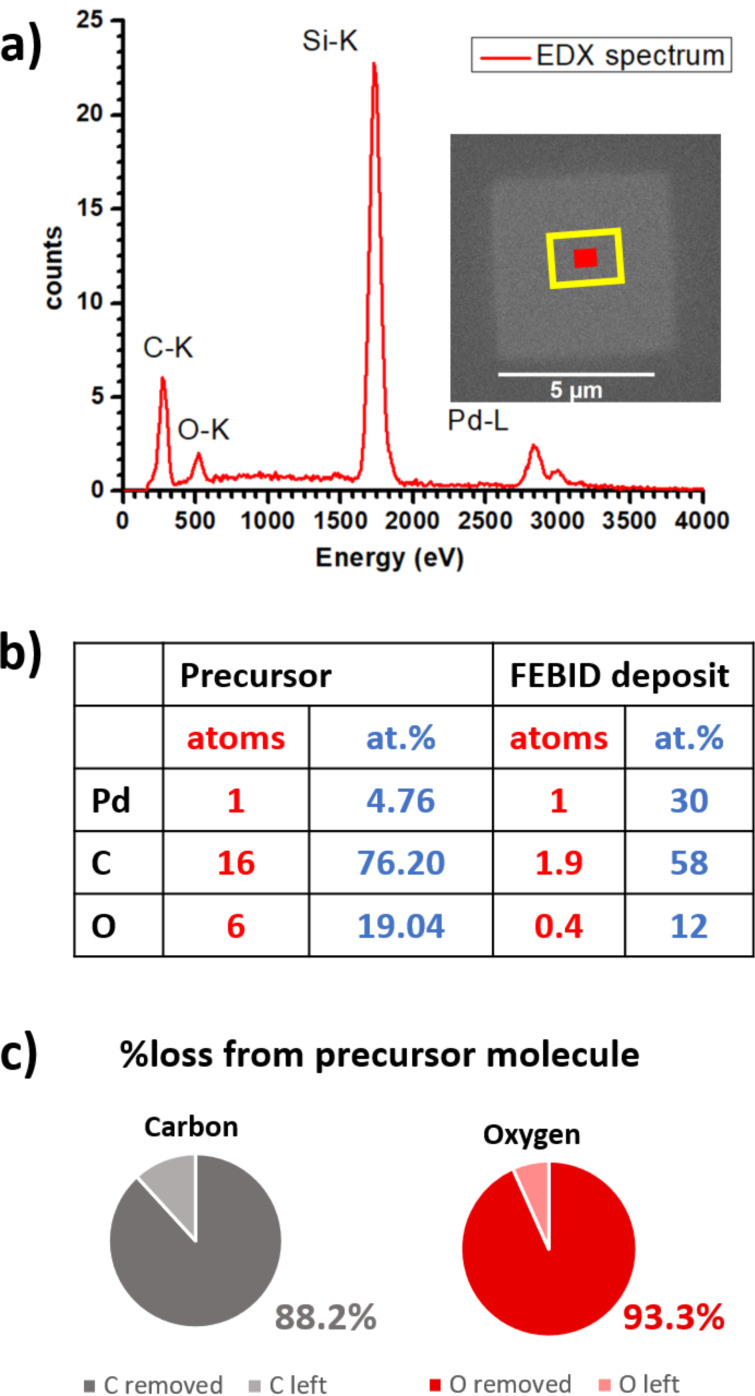
(a) EDX spectrum taken at the center of the deposit (red area) shown in inset. The BSE exit area is indicated with a yellow border. (b) Composition of the pristine [Pd(tbaoac)_2_] precursor and the FEB deposit (excluding H atoms). (c) Pie chart showing the element removal of carbon and oxygen from the intact precursor molecule during FEBID.

EDX measurements were conducted at the center of the deposit, marked as the red area in Figure 3a. The BSE range, calculated using the method of Kanaya and Okayama [46], is outlined with a yellow border. The prominent silicon peak indicates that thin film correction of EDX data is necessary for the accurate quantification of the thin Pd-containing FEB deposit. Using thickness values from AFM measurements and Stratagem thin film correction, the metal content was determined to be 30 atom %, see Figure 3b. Hydrogen was not included in the calculation as it cannot be detected by the EDX technique.

**Figure 4:**
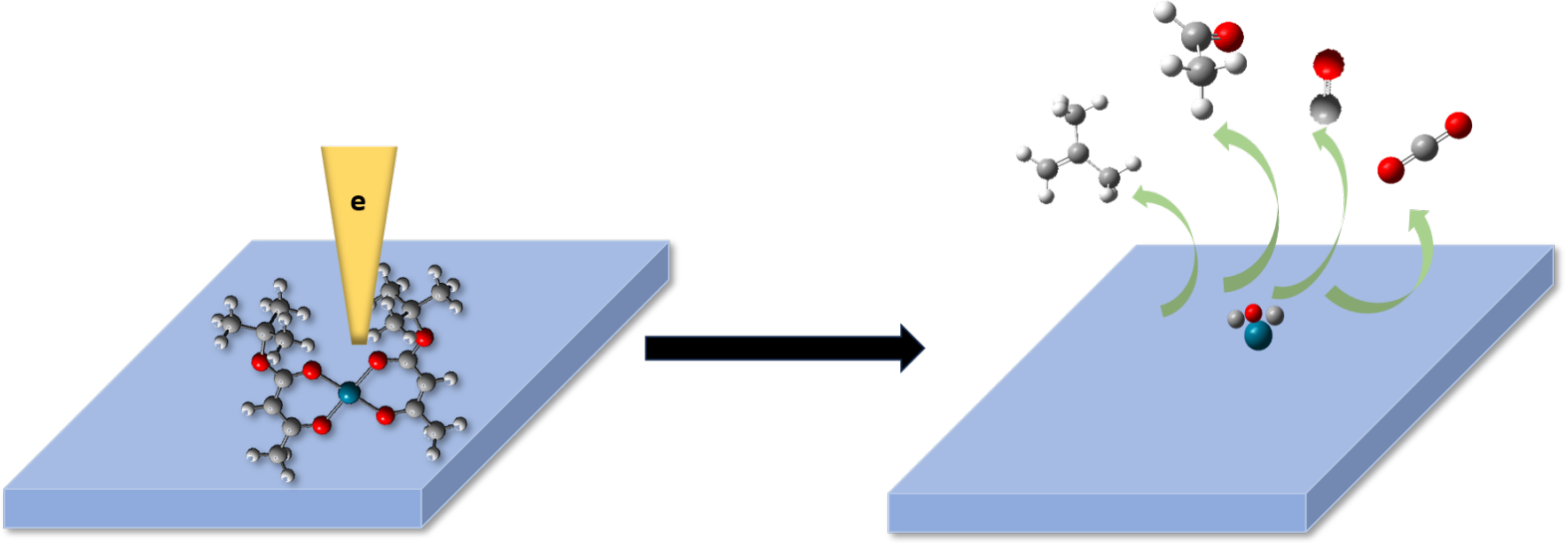
Illustration of the potential volatile fragments in the dissociation of adsorbed Pd(tbaoac)_2_ molecules yielding the observed composition of PdC_2_O_0.5_ of the FEBID material. Note that carbon co-deposition probably occurs from hydrocarbons. Hydrogen atoms cannot be quantified by EDX and are thus not illustrated here. The arrows symbolize the removal of the volatile fragments by the vacuum pump.

The total number of atoms in the pristine [Pd(tbaoac)_2_] precursor is 23 (excluding the 26 hydrogen atoms), which was reduced to about 3.3 atoms in the FEB deposit. Mass spectrometry studies of gas-phase electron-impact dissociation of [Pd(tbaoac)_2_] [42] showed the loss of *tert*-butyl (*t*-Bu) as (CH_3_)_2_CCH_2_ by dehydrogenation. Furthermore, the chelating ring collapse happens through the formation of O-containing molecular fragments such as CO, CH_3_CHO, and also CO_2_. These findings suggest that during FEB irradiation, the adsorbed [Pd(tbaoac)_2_] molecules may lose four carbon atoms per one (CH_3_)_2_CCH_2_ volatile molecular fragment, while the remaining carbon atoms may be removed as CO or CO_2_, hydrocarbons CH*_x_*, and other volatile species, such as CH_3_CHO, which were seen in gas phase dissociation experiments with this precursor [42], as illustrated in Figure 4.

Regardless of the exact stoichiometry of volatile species, it is noteworthy that from these large tbaoac ligands (each containing 8 C, 3 O, and 13 H atoms) about 90% of the carbon and oxygen atoms can be removed as shown in Figure 3c. This implies not only favorable bond dissociation channels resulting in volatile fragments, but also that the species desorb quickly before they (i) undergo further dissociation by electrons to form nonvolatile carbon products, (ii) polymerize with other fragments on the surface to form a nonvolatile matrix, or (iii) become embedded by newly arriving molecules from the gas phase [30]. A key factor for rapid desorption is the neutral charge of the generated fragments, which reduces the desorption energy. This applies to the isobutene, acetaldehyde, and carbon oxide fragments, making the tbaoac ligand favorable for FEBID.

Neglecting surface diffusion, the deposition rates ν_dep_ (in units of inverse time) and *R* (in units of distance over time) are given by [23]


[1]

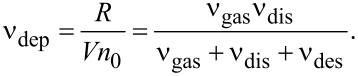



For the derivation of Equation 1 and for the numerical values used in the following, the reader is referred to Supporting Information File 1, section S2. From the composition, the molecular volume *V* of the decomposed molecule (PdC_2_O_0.5_) was roughly estimated to 5.3 × 10^−2^ nm^3^. Given the measured vertical growth rate of *R* = 0.02 nm·s^−1^, the deposition rate is ν_dep_ = 1.38 × 10^−1^ s^−1^ using the maximum monolayer coverage *n*_0_ = 2.72 nm^−2^ for the Pd(tbaoac)_2_ molecule. The gas adsorption rate is ν_gas_ = 1.36 × 10^3^ s^−1^, and the maximum dissociation rate is ν_dis_ = 6.16 × 10^3^ s^−1^. This gives the upper limit on the desorption rate of ν_des_ = 6.07 × 10^7^ s^−1^. The ratios ν_dis_/ν_des_ = 1.01 × 10^−4^ and ν_gas_/ν_des_ = 2.2 × 10^−5^ imply that deposition is performed in the desorption-driven (DD) regime [47]. The lower limit estimation of ν_des_ = 2 × 10^2^ s^−1^ implies the reaction rate-limited (electron-limited, EL) regime of deposition (see details in Supporting Information File 1, section S2). Interestingly, a fivefold increase in electron flux from 4 × 10^3^ to 2 × 10^4^ electrons·nm^−2^·s^−1^ (i.e., a current increase from 0.7 to 4.7 nA) in our experiments did not influence the composition within the EDX measurement error of about 5 atom %. However, a 4.5-fold increase in the growth rate was observed from the decrease in EDX silicon peak intensity (see Supporting Information File 1 in section S3). This is consistent with the expected proportional relationship between growth rate and electron flux in the DD regime and the EL regime. For the DD regime, ν_des_ ≫ (ν_gas_ + ν_dis_), and Equation 1 simplifies to ν_dep_ ≅ (ν_gas_/ν_des_)·ν_dis_. For the EL regime, ν_des_ + ν_gas_ ≫ ν_dis_, and Equation 1 simplifies to 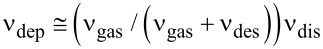. Both relations show the linear dependence of the FEBID growth rate on the dissociation rate, being itself proportional to the electron flux. Improving the estimations to decide on the deposition regime is complicated. In our case, considering the literature values for the electron interaction cross section, we tend to favor the DD regime conditions (please see details in Supporting Information File 1, section S2). However, in both regimes, molecular fragments will have enough time to desorb before being dissociated by the following incoming electrons.

The composition observed in FEBID was 30 atom % Pd, see Figure 3b. Looking at the 70 eV electron-impact gas-phase mass-spectrometry experiments [42] and assuming that the heavier Pd-containing fragments would remain on the surface during FEBID, the expected composition would be Pd/C/O = 1:7.2:4.3, corresponding to only 8 atom % Pd (see details in Supporting Information File 1, section S4). This highlights the importance of surface processes involved in achieving high metal contents during electron-induced dissociation including the role of SEs and BSEs. The FEBID deposit composition suggests that each [Pd(tbaoac)_2_] undergoes fragmentation leading to multiple fragments, either simultaneously or sequentially. Such a process requires a few electronvolts of energy deposited in the molecule, which hints towards the possible role of SEs (large energy transfer) and BSEs (multiple electron excitation) interacting with the molecule for efficient dissociation. Gas-phase mass-spectrometry experiments conducted using the same electron and molecular fluxes could provide further valuable insights to the role of surface processes when compared to FEBID experiments.

Of note is that we also performed a subtraction of the carbon contribution due to co-deposition from residual hydrocarbons, see details in Supporting Information File 1, section S5. Briefly, under our high-vacuum conditions, residual hydrocarbon gases lead to contamination deposits during both FEBID and EDX acquisition. To eliminate this contribution, we performed the same FEBID routine, however, without precursor gas, leaving only the residual gas. The EDX measurements were performed under the same conditions as the FEBID depositions, and the corresponding compositions were obtained (mainly carbon and oxygen). Stratagem thin film correction is applied for removing Si and SiO*_x_* as described in the Experimental section. From the measured composition, for the Pd FEBID deposit, the density is evaluated as the weighted average of Pd and C, and for all residual gas deposits having the composition C*_x_*O_1−_*_x_*, the density is assumed as 1.5 g·cm^−3^ [48]. AFM thickness measurements were used to determine the volume of the deposits and from there, the mass of the deposits was calculated. The individual numbers of O and C atoms in the residual gas FEB deposits and Pd precursor gas FEB deposits were calculated from their known weight percentages. Finally, the numbers of C and O atoms of the residual gas contribution are subtracted from those of the main FEB deposit from Pd precursor gas. This gives the contamination-corrected atomic percentages obtained by electron beam-induced deposition of the adsorbed precursor molecule under our experimental conditions. Using this procedure, we obtained a 2 atom % increase of Pd in the composition, namely 32 atom % Pd, 56 atom % C, and 12 atom % O or in atomic ratios Pd/C/O = 1:1.8:0.4. A density range of 1–3 g·cm^−3^ for residual gas deposits did not significantly alter the final composition of Pd, which remained within 32–34 atom %. It is important to note that this background subtraction may slightly overestimate the metal atom fraction because the surface is already partially covered by GIS-supplied molecules, potentially preventing residual hydrocarbon adsorption. Furthermore, surface reactions may be altered by the simultaneous presence of fragments of metalorganic and residual gas molecules. However, if there is plenty of carbonaceous matrix co-deposited by the metalorganic precursor molecules, one could hypothesize that the surface reactions of the residual gas molecules may be similar in type and yield to surface reactions of the residual gas molecules on its own deposit. A comparison with deposits made under UHV conditions in the future could be valuable for validating this method.

### Comparison among Pd precursors used in FEBID

The total number of atoms (excluding the hydrogen atoms) for the precursors [Pd(η^5^-Cp)(η^3^-allyl)], [Pd(hfac)_2_], and [Pd(tbaoac)_2_] are 9, 27, and 23 atoms respectively. The compositions in Table 1 show that the precursors [Pd(hfac)_2_] and [Pd(tbaoac)_2_] resulted in a composition similar to that from [Pd(η^5^-Cp)(η^3^-allyl)], though they contain more than double of the non-metal atoms. This fact highlights the important role of fluorine and oxygen atoms in the generation of volatile species under electron dissociation. It is important to note that the FEBID experiments using [Pd(η^5^-Cp)(η^3^-allyl)] and [Pd(hfac)_2_] were conducted with a field-emission gun and an electron energy of 1 keV. This lower electron energy increases the dissociation cross section and leads to greater heating of the deposit due to more energy deposited per unit trajectory length and, consequently, the small excitation volume where all the beam energy converts to heat. The related local temperature increase, especially for high beam currents and fine focus may have contributed to an increase in the metal content in the above cited experiments. Fluorine is well known for its etching properties and can efficiently remove carbon. The presence of oxygen can form volatile species such as CO_2_, CO, or alcohols. For [Pd(hfac)_2_], the breakage of the C–F bond was observed, and this is the main process responsible for fluorine removal [49].

**Table 1:** Highest reported FEBID Pd contents with different precursors. The substrate and GIS temperatures (*T*) are indicated. Note the highest ligand removal efficiency for the β-ketoesterate complex [Pd(tbaoac)_2_]. The arrows represent the reduction of atoms in the pristine molecule with respect to the number of atoms in the FEBID deposit composition. RT stands for room temperature.

Precursor	FEBID deposits atom %				% reduction from parent			Reference
*T*_substrate_/*T*_GIS_ °C	Pd	C	O	F	C	O	F	

[Pd(Cp)(allyl)] RT/30	29	71	—	—	68 (8→2.4)	—	—	[37]
[Pd(hfac)_2_] RT/30	24	52	11	13	78 (10→2)	88.5 (4→0.5)	95 (12→0.5)	[37]
[Pd(tbaoac)_2_] 70/85	30	58	12	—	88 (16→2)	93 (6→0.4)	—	this work

From an applicability standpoint, fluorine is less desirable because it can cause etching of other parts of the vacuum chamber where the deposition occurs. Since there are fewer carbon atoms in the deposit for [Pd(tbaoac)_2_] than for [Pd(η^5^-Cp)(η^3^-allyl)], the post-purification process to remove carbon can preserve better 3D shapes for the deposits made with [Pd(tbaoac)_2_]. Additionally, it is worth noting that substrates were held at room temperature for [Pd(η^5^-Cp)(η^3^-allyl)] and [Pd(hfac)_2_], which may explain the lower carbon removal efficiency due to residual gas contamination or less effective thermal desorption processes.

### Comparison with [Cu(tbaoac)_2_]

When comparing [Cu(tbaoac)_2_] and [Pd(tbaoac)_2_], the highest reported deposit metal content Cu/C/O was 1:2.3:0.5 [31], while for [Pd(tbaoac)_2_] in this work, the ratio Pd/C/O was 1:2:0.4, which are almost identical. However, considering the experimental conditions listed in Table 2, the substrate temperature was 30 °C higher for [Cu(tbaoac)_2_]. This increase in temperature likely enhances the desorption processes of organic ligand fragments and reduces residual gas deposition, improving the composition of the Cu content in the deposit.

**Table 2:** Comparison of [Pd(tbaoac)_2_] and [Cu(tbaoac)_2_] FEBID deposit compositions along with the experimental parameters employed.

Precursor	Temperature (°C)		Electron beam		C/M M = Pd, Cu	Metal atom %	Reference
	*T*_sub_	*T*_GIS_	*E* (keV)	e^−^ flux (electrons·nm^−2^·s^−1^)			

[Pd(tbaoac)_2_]	70	85	20	4 × 10^3^	1.8	30	this work
[Cu(tbaoac)_2_]	100	100	15	6 × 10^3^	2.3	26	[41]
[Cu(tbaoac)_2_]	134	120	20	1.5 × 10^3^	2.6	25	[50]
[Cu(tbaoac)_2_]	110	122	20	1.6 × 10^5^	4.1	17	[50]

Previous studies on [Pd(hfac)_2_] and [Cu(hfac)_2_], where [Pd(hfac)_2_] is in the singlet and [Cu(hfac)_2_] is in the doublet state, showed that [Pd(hfac)_2_] undergoes more extensive electron-induced fragmentation than [Cu(hfac)_2_], producing a greater variety of dissociation products [41]. This is because the electron attachment or ionization destabilizes [Pd(hfac)_2_] more than [Cu(hfac)_2_] [51]. We consider that the similar situation holds for [Cu(tbaoac)_2_] and [Pd(tbaoac)_2_] because they are also in the doublet and singlet states, respectively. Therefore, we anticipate a lower metal content for [Cu(tbaoac)_2_] than for [Pd(tbaoac)_2_] deposits. Furthermore, [Cu(tbaoac)_2_] produces comparatively larger fragments, see the mass spectrum from Devi et al. [40], which generally have lower desorption rates.

The volume deposition rate reported for [Cu(tbaoac)_2_] was 0.026 µm^3^·nA^−1^·min^−1^ [41], while for [Pd(tbaoac)_2_], it is 0.045 µm^3^·nA^−1^·min^−1^. The higher deposition rate for [Pd(tbaoac)_2_] implies that this precursor has potential for growing high-aspect ratio nanopillars as reported for [Cu(tbaoac)_2_] [31].

### Nanopillar with [Pd(tbaoac)_2_]

**Figure 5:**
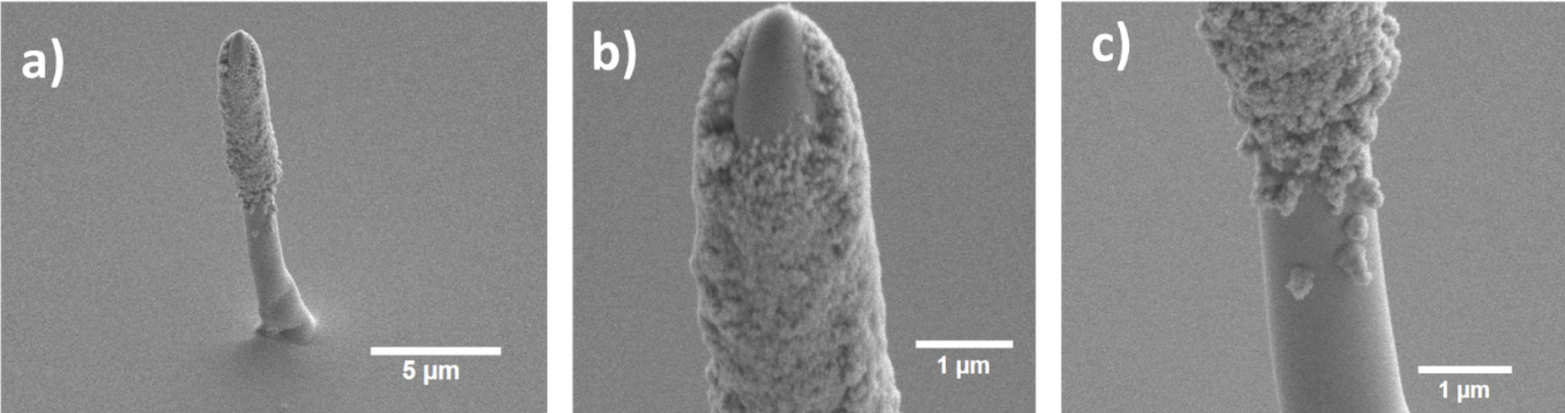
(a) Nanopillar grown by [Pd(tbaoac)_2_]-assisted FEBID. (b) Magnified view of the nanopillar tip. (c) The region where the growth transition to thermal molecular dissociation at the pillar walls (grainy shell structure) due to local electron beam heating. All the images were taken at a tilt angle of 45°.

A nanopillar was grown with [Pd(tbaoac)_2_] by FEBID and a beam current of 700 pA at 20 keV with a substrate temperature of 70 °C and a GIS temperature of 85 °C, as shown in Figure 5. We observed the formation of a granular deposit at the upper part of the pillar. Similar observations have been reported in a detailed previous study with a dimethyl(acetylacetonate)gold precursor and were attributed to the thermal decomposition of the precursor at the pillar apex due to local heating by energy implantation from the primary electron beam [52]. The deposition rate is 0.23 nm·s^−1^. The volume growth rate for the nanopillar neglecting the granular deposit would be 0.019 µm^3^·min^−1^, while for the square deposit in Figure 2a it is 0.032 µm^3^·min^−1^. In general, we expect the volume growth rate to be identical given the same molecular and electron flux. However, for the pillar, the surface diffusion contribution of adsorbates is lower as the diffusing molecules need to travel a longer distance from the surface to reach the irradiated area. Also, molecules are lost because of thermal decomposition that is occurring on the pillar walls in the present case, eventually causing lower volume growth rate for pillars. The tip of the high-aspect ratio pillar follows the electron beam profile without any depletion at the center, further demonstrating the potential of the [Pd(tbaoac)_2_] precursor for creating 3D nanostructures in FEBID applications.

## Conclusion

In this study, we investigated palladium deposits obtained with [Pd(tbaoac)_2_], having non-fluorinated β-ketoesterate ligands, in the FEBID process. Under the given experimental conditions, square deposits exhibited a central dip, indicating a significant surface diffusion process taking place. TEM analysis revealed metal nanograins embedded in a carbonaceous matrix. The deposits contained up to 30 atom % Pd, with a prominent reduction of about 90% of the 23 carbon atoms in the precursor. Using a continuum model, the calculated upper limit of the desorption rate shows that the deposits were primarily formed in the desorption-driven regime, while the lower limit of the desorption rate gives the electron-limited regime. However, we favor the desorption-driven regime prevailing in our experiments. To correct for residual gas contamination, we applied a method that could be broadly used for any FEBID deposits. In our case, the carbon contamination contributed 2–4 atom % carbon content in the FEB deposit. The [Pd(tbaoac)_2_] precursor exhibits the highest reduction in carbon content by FEBID compared to other precursors with large ligands, highlighting the importance of further studies of dissociation and desorption mechanisms involved in the removal of the carbon- and oxygen-rich ketoesterate ligands. The nanopillar formed from this precursor demonstrates its potential for 3D nanostructure fabrication using FEBID.

## Supporting Information

File 1 Additional experimental details and details on the theoretical calculations.

## Data Availability

Data generated and analyzed during this study is available from the corresponding author upon request.
