# Supplementary material for: Electron beam-based direct writing of nanostructures using a palladium β-ketoesterate complex
Source: Beilstein J Nanotechnol. 2025 Apr 15;16:530–9. doi: 10.3762/bjnano.16.41 (PMC12018897; doi:10.3762/bjnano.16.41)
Supplement: File 1 — Additional experimental details and details on the theoretical calculations. [file Beilstein_J_Nanotechnol-16-530-s001.pdf]

BEILSTEIN JOURNAL OF NANOTECHNOLOGY

Supporting Information

for

Electron beam-based direct writing of nanostructures using a palladium β-ketoesterate complex

Chinmai Sai Jureddy, Krzysztof Maćkosz, Aleksandra Butrymowicz-Kubiak, Iwona B. Szymańska, Patrik Hoffmann and Ivo Utke

Beilstein J. Nanotechnol. **2025,** *16,* 530–539. doi:10.3762/bjnano.16.41

Additional experimental details and details on the theoretical calculations

License and Terms: This is a supporting information file under the terms of the Creative Commons Attribution License (https://creativecommons.org/licenses/by/4.0). Please note that the reuse, redistribution and reproduction in particular requires that the author(s) and source are credited and that individual graphics may be subject to special legal provisions.
The license is subject to the *Beilstein Journal of Nanotechnology* terms and conditions: (https://www.beilstein-journals.org/bjnano/terms)

S1 Scanning transmission electron microscope (STEM) images

For better visualization of crystalline structure, the standalone images in Figure 2e,f of the main manuscript are presented in Figure S1a1 and Figure S1b, respectively. The atomic columns oriented along crystallographic directions in the grains can be clearly observed in Figure S1a1; additionally another STEM image is shown in Figure S1a2. The labeling of the diffraction pattern from the selected area electron diffraction (SAED) measurements is presented in Figure S1b.

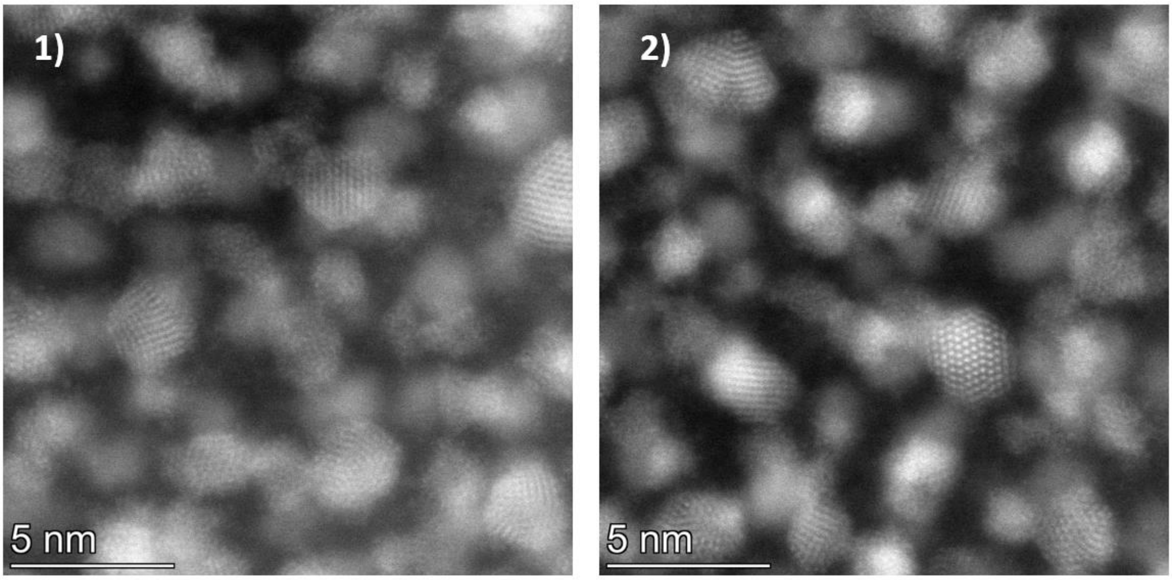

Figure S1a: 1) The standalone image of Figure 1e in the main manuscript. It is a HR-STEM image from the edge of the deposit in Figure 1c. 2) Additional STEM image. The crystalline structure of the grains and the atomic columns oriented along crystallographic directions can be observed.

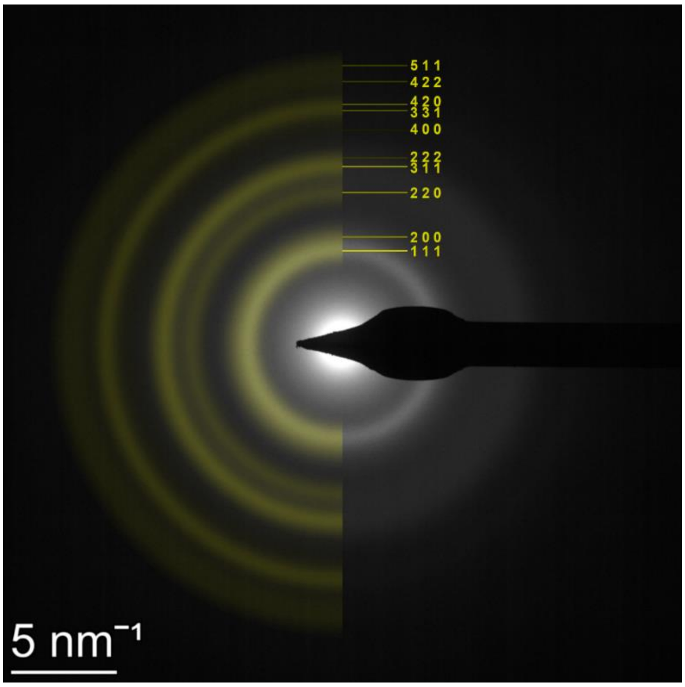

Figure S1b: SAED pattern from the edge of the deposit in Figure 1c.

S2 Parameters used in desorption rate calculation

Neglecting the surface diffusion, the continuum model for the variation of the adsorbed molecular surface density, n, can be written as,

$$\frac{dn}{dt} = sJ\left(1 - \frac{n}{n_0}\right) - \frac{n}{\tau} - \sigma n f \qquad \text{(S2a)}$$

The definitions of labels used are reported in Table S2. Under steady-state conditions, Equation S2a can be solved for n as

$$n = \frac{sJ}{\frac{sJ}{n_0} + \frac{1}{\tau} + \sigma f} \qquad \text{(S2b)}$$

The vertical growth rate, $R = nV\sigma f$, using Equation S2a, becomes

$$R = \frac{sJ}{\frac{sJ}{n_0} + \frac{1}{\tau} + \sigma f} V\sigma f \qquad \text{(S2c)}$$

Introducing the deposition rate, $\nu_{dep} = R/Vn_0$, the desorption rate, $\nu_{des} = 1/\tau$, the dissociation rate, $\nu_{dis} = \sigma f$, and the gas impingement rate $\nu_{gas} = sJ/n_0$, Equation S2c can be rewritten as

$$\nu_{dep} = \frac{R}{Vn_0} = \frac{\nu_{gas}\nu_{dis}}{\nu_{gas} + \nu_{dis} + \nu_{des}} \qquad \text{(S2d)}$$

The symbols are described in Table S2.

Equation S2d can be rearranged for estimating the desorption rate ν_{des} as follows,

$$\nu_{des} = \frac{\nu_{gas}\nu_{dis}}{\nu_{dep}} - (\nu_{gas} + \nu_{dis}) \qquad \text{(S2e)}$$

Substituting the definitions of the rates, Equation S2e can be written as,

$$\nu_{des} = \sigma f\left(\frac{VsJ}{R} - 1\right) - \frac{sJ}{n_0} \qquad \text{(S2f)}$$

The quantities J, R, and f are determined experimentally. The volume of the decomposed molecule, $V(PdC_2O_{0.5})$ is calculated using the relation $V = M_{PdC2O0.5}/\rho N_A$. The density ρ is obtained by rewriting the composition as $Pd_{0.29}C_{0.57}O_{0.14}$. The density can then be calculated as the weighted average density according to Utke et al. [1] as $\rho = 0.29\rho_{Pd} + 0.57\rho_C$ with $\rho_{Pd} = 12.007$ cm^{-3} and taking $\rho_C = 1.5$ g/cm^3, which is the density of amorphous hydrogenated carbon. $M_{PdC2O0.5}$ is the molar mass of the molecule ($M_{PdC2O0.5} = 138.42$ g/mol), and N_A is the Avogadro number. The volume of the decomposed molecule becomes $V = 5.3\cdot10^{-2}$ nm^3.

The monolayer density (n_0) of the [Pd(tbaoac)$_2$] molecule adsorbing on the substrate surface is calculated as $1/\pi r^2$, *r* being the radius of the adsorbing molecule, or as $1/A$, *A* being the projected area of the adsorbed molecule on the surface. The fragmentation cross section under electron impact is σ. The quantities σ and n_0 are unknown and need to be estimated through logical reasoning. They can be used to set the upper and lower limit on ν_{des} given by Equation S2f.

First, we estimate the upper limit value. The maximum value of σ can be considered as the geometrical cross section of the molecule, which is 1.54 nm^2, considering the molecular size (diameter) as roughly 1.4 nm. The maximum value of n_0 occurs for the configuration shown in Figure S2b, with the molecule being adsorbed in an upright fashion. This corresponds to a projected area of 0.368 nm^2 and n_0 = 2.72 nm^{-2}. Using these values, ν_{dep} = 1.38·10^{-1} s^{-1}, ν_{gas} = 1.36·10^3 s^{-1} (assuming a sticking coefficient of 1), ν_{dis} = 6.16·10^3 s^{-1}, and the upper limit estimated value of ν_{des} = 6.07·10^7 s^{-1}. These values are tabulated in Table S2. The ratios ν_{dis}/ν_{des} = 1.01·10^{-4} and ν_{gas}/ν_{des} = 2.2·10^{-5} are very small compared to unity and imply that the desorption driven regime prevails.

For the lower limit of ν_{des}, the minimum coverage can be estimated by considering the molecule to be adsorbed completely flat (Figure S2c). This gives n_0 = 0.9 nm^{-2}. For the lower limit on σ, we consider the fact that the rates are non-negative. The right-hand side of the Equation S2f should be greater than or equal to zero. This gives the lower limit on σ. The equations are derived as follows,

$$\nu_{des} = \sigma f\left(\frac{VsJ}{R} - 1\right) - \frac{sJ}{n_0} \geq 0 \quad \text{(S2g)}$$

Rearranging Equation S2g gives,

$$\sigma \geq \frac{sJR}{fn_0(sJV - R)} \qquad \text{(S2h)}$$

Using the value of n_0 = 0.9 nm^{-2} gives $\sigma \geq$ 1.1·10^{-4} nm^2 = 0.011Å^2. Both values translate to ν_{dep} = 4.19·10^{-1} s^{-1}, ν_{gas} = 4.1·10^3 s^{-1}, ν_{dis} = 4.4·10^{-1} s^{-1}, and the lower limit estimated value of ν_{des} = 2·10^2 s^{-1}. With these values, only the ratio ν_{dis}/ν_{des} = 2.2·10^{-3} is smaller than unity, while the ratio ν_{gas}/ν_{des} = 2.05·10^1 is larger than unity. This would imply that the deposition proceeds in the reaction-rate limited (electron-limited) regime. When we performed deposition with high electron flux, using the upper limit estimation of ν_{des}, it is consistent with the expected relationship between growth rate and electron flux in the desorption-driven regime. Since, $\nu_{des} \gg (\nu_{gas} + \nu_{dis})$,

Equation S2d simplifies to $\nu_{dep} \cong (\nu_{gas}/\nu_{des.}) \cdot \nu_{dis}$ showing a linear dependence on the dissociation rate being itself proportional to the electron flux. When $\nu_{gas} + \nu_{des} \gg \nu_{dis}$, then Equation S2d simplifies to $\nu_{dep} \cong \left(\frac{\nu_{gas}}{\nu_{gas}+\nu_{des}}\right)\nu_{dis}$, which is also proportional to ν_{dis}. It follows that the growth regime can be either desorption-driven or electron-limited. If we consider $\sigma = 1.1{\cdot}10^{-3}$ nm^2, then the deposition regime is in transition to desorption driven regime (ν_{gas}/ν_{des} = 1.05, for $\sigma = 1.1{\cdot}10^{-3}$ nm^2). The literature values reported in Table VII of Utke et al. [2] shows that at 100 keV, the FEB contamination deposit has cross sections ranging from 0.007 to 0.02 nm^2. We have lower electron energy, and we expect the cross section would be higher at 20 keV. As the contamination deposit is primarily due to residual hydrocarbons, we think [Pd(tbaoac)$_2$] will have a higher electron impact cross section than hydrocarbons in residual gases given its higher molecular size. This can observed for example in the paper of Schram et al. 1996 [3], where the ionization cross section increases with increasing size of molecules. Therefore, we think that the deposition process takes place more probably in the desorption-driven regime.

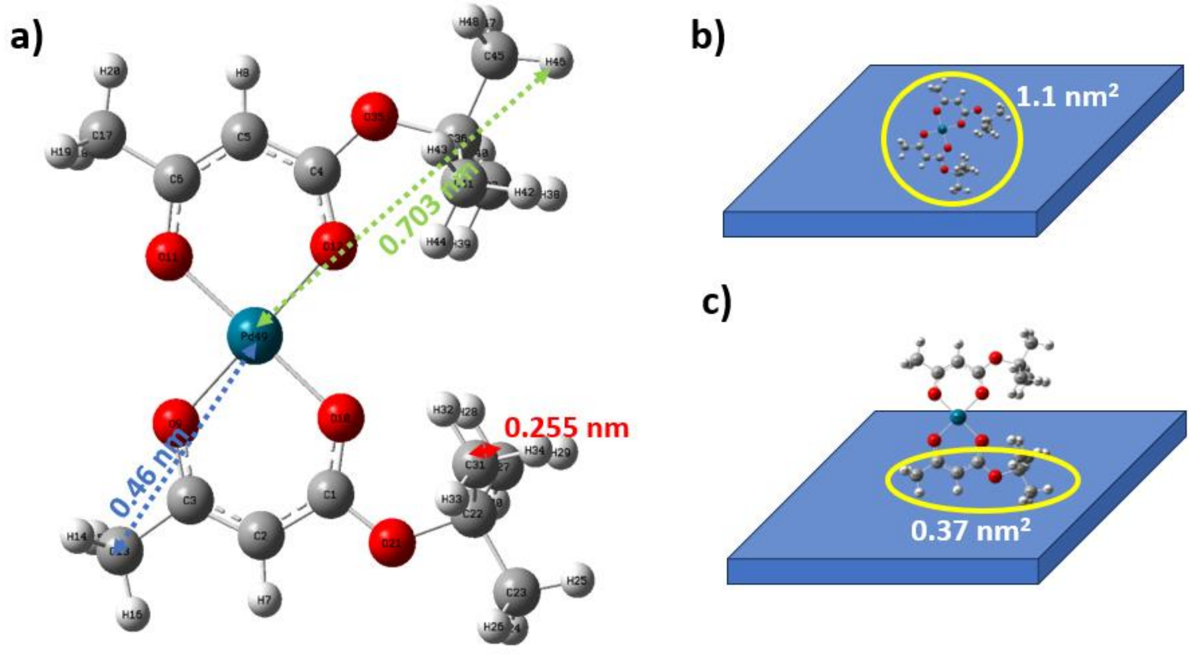

Figure S2: (a) DFT-optimized structure of [Pd(tbaoac)$_2$], calculated using the basis set LanL2DZ for Pd and 6-311+(d,p) for C,O,H atoms and with B3LYP correlation function in Gaussian software [4]. The distances between the atoms are represented with green arrows and corresponding length is reported. (b) The flat adsorption geometries of [Pd(tbaoac)$_2$] on a substrate, which gives minimum monolayer surface density. The projected area is marked and reported. (c) The same as (b), but with upright configuration giving maximum monolayer surface density.

Table S2: Parameters entering into Equation S2 and values used for the calculation of the desorption rate ν_{des} for the square deposit in Figure 2a. The values from experimental measurements are highlighted in blue.

Deposition Rate R	R ($nm \cdot s^{-1}$)	0.02
Volume of decomposed molecule	V (nm^3)	$5.3 \cdot 10^{-2}$
Monolayer surface density	n_0 (nm^{-2})	2.72
Deposition rate ν_{dep}	$\nu_{dep} = R/Vn_0$ (s^{-1})	$1.38 \cdot 10^{-1}$
Precursor flux	J ($molecules \cdot nm^{-2} \cdot s^{-1}$)	$3.7 \cdot 10^3$
Sticking coefficient	s	1
Gas impingement rate	$\nu_{gas} = sJ/n_0$ (s^{-1})	$1.36 \cdot 10^3$
Electron flux	f ($electrons \cdot nm^{-2} \cdot s^{-1}$)	$4 \cdot 10^3$
Dissociation cross section	σ (nm^2)	1.54
Dissociation rate	$\nu_{dis} = \sigma f$ (s^{-1})	$6.16 \cdot 10^3$
Desorption rate	$\nu_{des} = \frac{\nu_{gas}\nu_{dis}}{\nu_{dep}} - (\nu_{gas} + \nu_{dis})$	$6.07 \cdot 10^7$

S3 Deposit with high electron flux

Figure S3 compares deposits grown with two different electron fluxes. Within the EDX measurement uncertainty of ±5 atom %, the composition of both deposits can be considered the same. No carbon background subtraction was performed. Note the composition of the deposit at higher electron flux is reported without thin film correction.

electron flux of 1.86×10^4 electrons/nm^{2}s

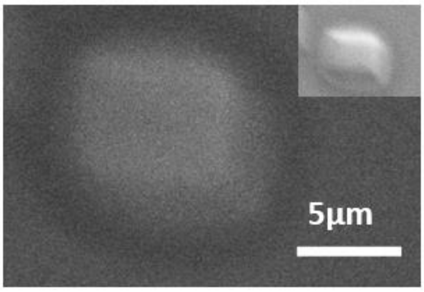

	at.% without thin film correction
Pd	24
C	67
O	9
Si	0.7

electron flux of 3.84×10^3 electrons/nm^{2}s

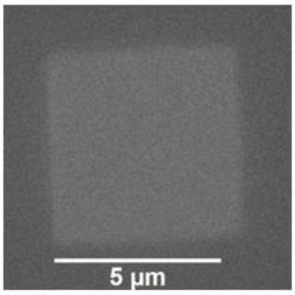

	at.% without thin film correction	at.% after thin film correction
Pd	9	30
C	36	58
O	4	12
Si	51	-

Figure S3: SEM images of FEB deposits obtained with two different electron fluxes. The inset on the left image is the 30° tilted view of the deposit. The corresponding composition were tabulated under the image with and without thin film correction. Note the difference in the Si atom % value, which is due to different deposit thicknesses (the thicker the deposit the less is the Si atom % value). Observing how the Si content for the right deposit is distributed to other elements after thin film correction, we can expect the Pd content for the left deposit can at most increase to 25 atom % after the correction.

S4 Estimation of composition from gas phase experiments

Table S4 shows the relative intensities of Pd-containing fragments obtained in the 70 eV electron impact dissociative ionization of the gas phase molecule [Pd(tbaoac)$_2$] [5]. The number of Pd, C, and O atoms were calculated in each fragment and multiplied to the relative intensity of the corresponding fragment. Assuming these fragments stay on the surface, the total numbers of Pd, C, and O atoms (N_{Pd}, N_C, and N_o) were obtained by summing the contribution from all fragments. Then the metal content is estimated as N_{Pd}/N_{total}; $N_{total} = N_{Pd} + N_C + N_o$, which is 8 atom %.

Table S2: Relative intensity of Pd-containing fragments from electron impact gas phase dissociation and the numbers of Pd, C, and O atoms that each fragment contributes.

Fragment	No of atoms in the fragment			Relative intensity	No of atoms after multiplying intensity		
	Pd	C	O		Pd	C	O
[Pd(CH$_3$COCH$_2$)]$^+$	1	3	1	20	20	60	20
[Pd(CH$_3$COCHCO)]$^+$	1	4	2	32	32	128	64
[Pd(CH$_3$COCH$_2$CO$_2$H)]$^+$	1	4	3	64	64	256	192
[Pd(CH$_3$COCH$_2$CO$_2{}^t$Bu)]$^+$	1	8	3	9	9	72	27
[Pd(CH$_3$COCH$_2$CO$_2$)$_2$]$^{+\cdot}$	1	8	6	100	100	800	600
[Pd(CH$_3$COCHCO$_2{}^t$Bu)(CH$_3$COCH$_2$CO$_2$)]$^{+\cdot}$	1	12	6	11	11	132	66
[Pd(CH$_3$COCHCO$_2{}^t$Bu)$_2$]$^{+\cdot}$	1	16	6	30	30	480	180
				Total	N_{Pd} = 266	N_C = 1928	N_O = 1149

S5 Subtraction of residual gas contribution to the composition

Residual gases present in the SEM can adsorb on the surface and get co-deposited along with the main precursor gas in FEBID. This co-deposition often overestimates the carbon content, which can be seen through the contrast observed in the SEM imaging area. We try to subtract such residual gas co-deposition through the methodology shown in Figure S5a,b. Figure S5a shows the general idea of removing the residual gas contribution by performing the residual gas deposition under the same conditions as the precursor FEBID and then subtracting its contribution from the precursor FEBID. The composition of these deposits is obtained through EDX measurements with the same measurement parameters. AFM measurements were done to obtain the volume of the deposit. From the composition, the density was calculated, and the mass of the deposit was obtained from the known volume of the deposit. Then the mass of the deposit is used to calculate the number of C and O atoms in the deposit from their weight percentages. The contamination-free deposit composition is obtained by subtracting the residual gas deposit C and O atoms from

the C and O atoms of the precursor deposit. The schematic of the methodology to subtract the residual gas contamination is shown in Figure S5b.

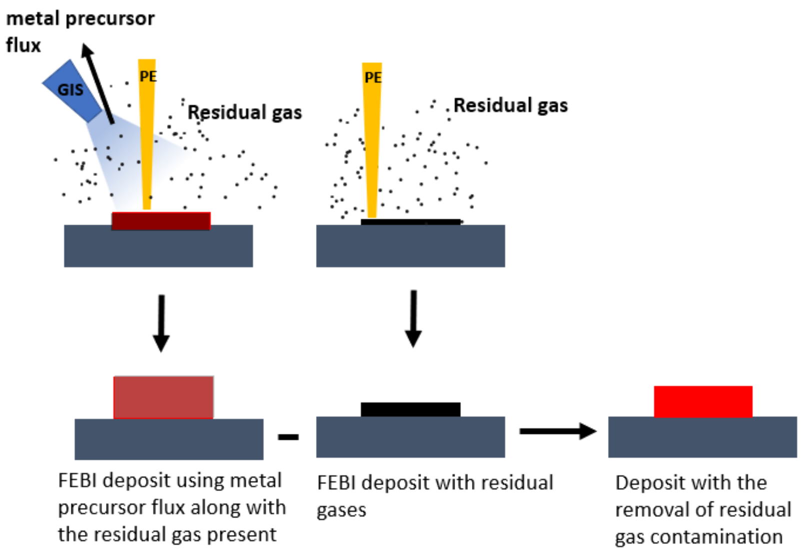

Figure S5a: Schematic showing the FEBID experiments needed for experimental separation of the co-deposited carbon contribution (from residual gas or other carbon sources) in the EDX quantification. FEB depositions are performed with precursor flux (red) and residual gas (black) and with residual gas alone under the same conditions. The composition of these deposits is measured by EDX using identical EDX parameters and allows one to subtract the carbon content co-deposited by the presence of residual gas, also see Figure S3.

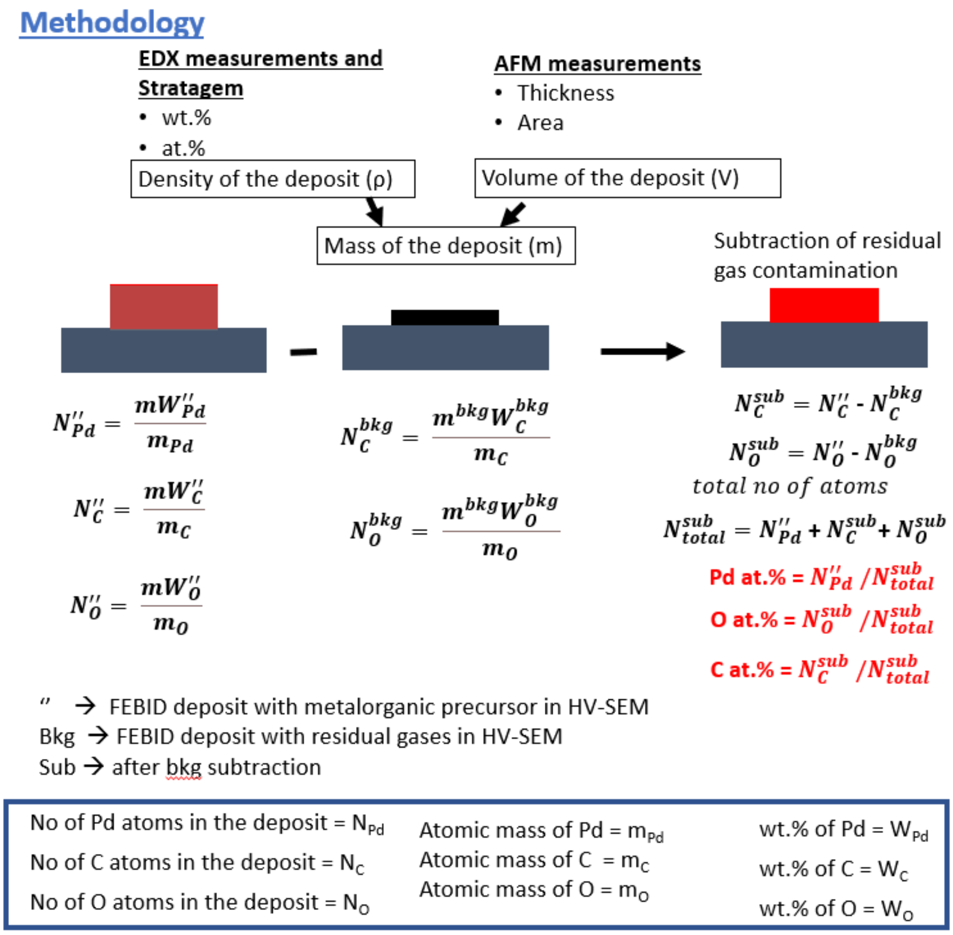

Figure S5b: Methodology to remove the residual gas contribution from the precursor FEB deposit by combined EDX with Stratagem thin film correction (deposit density and deposit composition) and AFM measurements (thickness, area, and volume) enabling the necessary calculation of the number of atoms in the deposits.

Application of the method to the FEBID using [Pd(tbaoac)$_2$]

The residual gas depositions used for the subtraction procedure were performed at substrate temperatures of 50 and 40 °C. The residual gas deposition with T_{sub} = 70 °C was too thin to be reliably measured by AFM. Therefore, an appropriate extrapolation of the number of atoms was performed from the 40 and 50 °C contamination deposits. The depositions were made with an electron flux of $2 \cdot 10^4$ electrons·nm^{-2}·s^{-1}, and linear scaling was applied for the deposition time. For the FEBID conditions of the Pd–C deposit reported in Figure 2 in the main manuscript, the residual gas deposit is estimated to contain $4.4 \cdot 10^8$ C atoms·µm^{-2} and $0.74 \cdot 10^7$ O atoms·µm^{-2}. The composition of the Pd–C FEB deposit amounts to $2.9 \cdot 10^9$ Pd atoms·µm^{-2}, $5.5 \cdot 10^9$ C atoms·µm^{-2}, and $1.2 \cdot 10^9$ O atoms·µm^{-2}. After the subtraction of C and O atoms of the residual gas deposit, the contamination-corrected composition gave 32 atom % Pd, 55 atom % C, and 13 atom % O. Depending on the assumed density of the background contamination deposit (1–3 g·cm^{-3}), the carbon background

correction resulted in 2–4 atom % more Pd content with respect to Figure 3b in the main text.

References

(1) Utke, I.; Friedli, V.; Michler, J.; Bret, T.; Multone, X.; Hoffmann, P. Density Determination of Focused-Electron-Beam-Induced Deposits with Simple Cantilever-Based Method. *Appl. Phys. Lett.* **2006**, *88* (3), 031906. https://doi.org/10.1063/1.2158516.

(2) Utke, I.; Hoffmann, P.; Melngailis, J. Gas-Assisted Focused Electron Beam and Ion Beam Processing and Fabrication. *J. Vac. Sci. Technol. B Microelectron. Nanometer Struct. Process. Meas. Phenom.* **2008**, *26* (4), 1197–1276. https://doi.org/10.1116/1.2955728.

(3) Schram, B. L.; Van Der Wiel, M. J.; De Heer, F. J.; Moustafa, H. R. Absolute Gross Ionization Cross Sections for Electrons (0.6–12 keV) in Hydrocarbons. *J. Chem. Phys.* **1966**, *44* (1), 49–54. https://doi.org/10.1063/1.1726502.

(4) Frisch, M. J.; Trucks, G. W.; Schlegel, H. B.; Scuseria, G. E.; Robb, M. A.; Cheeseman, J. R.; Scalmani, G.; Barone, V.; Petersson, G. A.; Nakatsuji, H.; Li, X.; Caricato, M.; Marenich, A. V.; Bloino, J.; Janesko, B. G.; Gomperts, R.; Mennucci, B.; Hratchian, H. P.; Ortiz, J. V.; Izmaylov, A. F.; Sonnenberg, J. L.; Williams; Ding, F.; Lipparini, F.; Egidi, F.; Goings, J.; Peng, B.; Petrone, A.; Henderson, T.; Ranasinghe, D.; Zakrzewski, V. G.; Gao, J.; Rega, N.; Zheng, G.; Liang, W.; Hada, M.; Ehara, M.; Toyota, K.; Fukuda, R.; Hasegawa, J.; Ishida, M.; Nakajima, T.; Honda, Y.; Kitao, O.; Nakai, H.; Vreven, T.; Throssell, K.; Montgomery Jr., J. A.; Peralta, J. E.; Ogliaro, F.; Bearpark, M. J.; Heyd, J. J.; Brothers, E. N.; Kudin, K. N.; Staroverov, V. N.; Keith, T. A.; Kobayashi, R.; Normand, J.; Raghavachari, K.; Rendell, A. P.; Burant, J. C.; Iyengar, S. S.; Tomasi, J.; Cossi, M.; Millam, J. M.;

Klene, M.; Adamo, C.; Cammi, R.; Ochterski, J. W.; Martin, R. L.; Morokuma, K.; Farkas, O.; Foresman, J. B.; Fox, D. J. Gaussian 16 Rev. C.01, 2016.

(5) Butrymowicz-Kubiak, A.; Muzioł, T. M.; Kaczmarek-Kędziera, A.; Jureddy, C. S.; Maćkosz, K.; Utke, I.; Szymańska, I. B. New Palladium(II) β-Ketoesterates for Focused Electron Beam Induced Deposition: Synthesis, Structures, and Characterization. *Dalton Trans.* **2024**, *53* (32), 13662–13677. https://doi.org/10.1039/D4DT01287A.
